# Discrimination between Leave of *Apocynum venetum* and Its Adulterant, *A. pictum* Based on Antioxidant Assay and Chemical Profiles Combined with Multivariate Statistical Analysis

**DOI:** 10.3390/antiox4020359

**Published:** 2015-05-08

**Authors:** Chi-On Chan, Ching-Ching Lau, Yam-Fung Ng, Li-Jia Xu, Si-Bao Chen, Shun-Wan Chan, Daniel Kam-Wah Mok

**Affiliations:** 1 State Key Laboratory of Chinese Medicine and Molecular Pharmacology, Department of Applied Biology and Chemical Technology, The Hong Kong Polytechnic University, Shenzhen 518057, China; E-Mails: on.chan@polyu.edu.hk (C.-O.C.); sw.chan@polyu.edu.hk (S.-W.C.); 2 Department of Applied Biology and Chemical Technology, The Hong Kong Polytechnic University, Hong Kong, China; E-Mails: christy.lau@connect.polyu.hk (C.-C.L.); yam-fung@hotmail.com (Y.-F.N.); 3 The Institute of Medicinal Plant Development, Chinese Academy of Medical Sciences and Peking Union Medical College, Beijing, 100193, China; E-Mail: xulijia@hotmail.com

**Keywords:** *Apocynum venetum* L., *Apocynum pictum* Schrenk, HPLC fingerprints, free radical scavenging capacity

## Abstract

An integrated approach including chemical and biological assessments was developed to investigate the differences between *Apocynum venetum* L*.* (AV) and its adulterant, *Apocynum pictum* Schrenk (AP). Ten flavonoids were tentatively identified by ultra-visible and mass spectra data. The chemical component, hyperoside, was identified as a critical parameter for discrimination of two species from the results of principal component analysis (PCA) and quantitative analysis. The anti-oxidative power of the herbal extracts were determined using 2,2-diphenyl-1-(2,4,6-trinitrophenyl) hydrazyl (DPPH) assay and H_2_O_2_-induced cell damage on LO2 cells. The results of the biological assays suggested that the chemical differences between AV and AP do lead to difference in activity and AV is demonstrated to have higher anti-oxidant activity.

## 1. Introduction

Traditional Chinese medicines (TCMs) have attracted global attention for their various applications in therapeutic agents or diet supplements; the increasing trend of consuming TCMs has resulted in a serious resource problem. Therefore, related plant species from the same genus are often used as substitute in many folk medicine practices assuming similar species usually contain the same classes of compounds and similar therapeutic activities. Although the species from same genus may contain the same classes of compounds, the detailed chemical components present are very often different. A comparison of their chemical composition and pharmacological activities is certainly needed [1] to support the use of an alternative species. In this study, the chemical composition and biological activities of two related species *Apocynum venetum* L. (AV) and *A. pictum* Schrenk (AP) are studied to compare their similarity and differences in chemical composition and biological activities.

*Apocynum venetum* L. (AV), (*Luobuma* in Chinese, *Apocynaceae*), is a perennial herbaceous or half-shrub plant that grows ubiquitously in central to northwestern China [2]. Its leave have a long history as a Chinese traditional medicine with uses to calm the liver, soothe the nerves, dissipate heat, and promote dieresis [3]. Previous studies reported that AV extract exhibits a series of pharmacological activities, such as anti-oxidation [4,5], anti-hypertensive [6,7] and anti-depressant effects [8,9], cholesterol-lowering [10] and AGEs-inhibitory activity [5], as well as its main active fractions were found to be phenolic acid, flavones and flavan-3-ols components [11,12]. As a result, AV has been applied and appeared to be a popular daily beverage in China, Japan and the US [13]. Various formulations of this herb, including tea bags, powder, and crimped and curled roasted leaves, have recently been commercialized as a sedative and anti-aging supplement, which has become increasingly popular in North American and East Asian health food markets [14].

*A. pictum* (AP), an easily confusable herb for AV, is similar to AV in terms of plant morphological characteristics and geographical distribution. Generally, AP is known locally as White Hemp, while AV is known as Red Hemp, probably due to the different colors and shapes of their flowers and leaves. The industrial applications of AP are limited to fibers for spinning and papermaking rather than medicinal purposes [13]. Due to the excessive exploitation, wild AV has dropped in recent years even though some protective measures have been enacted [15]. Because of the scarcity of AV, and since AP has a rather similar appearance to AV, AP is very often used as a substitute in the market. In order to distinguish both species and guarantee the quality of products derivated from this herb, the present study establishes an integrative approach to investigate the differences of chemical constituents and antioxidant activities between AV and AP. High performance liquid chromatography linked with diode array detection and mass spectrometer was applied to identify the flavonoids presented in AV and AP. The anti-oxidative capacity of the extracts was also determined using chemical (DPPH test) and biological (*in vitro* H_2_O_2_-induced cell model) methods.

## 2. Experimental Section

### 2.1. Chemicals and Reagents

Reference standards of hyperoside and isoquercetrin, (purity > 98%) were obtained from the National Institute for the Control of Pharmaceutical and Biological Products (Beijing, China). Analytical grade formic acid and trifluoroacetic acid were purchased from Wako (Japan) and Aldrich (USA), respectively, while HPLC grade acetonitrile was obtained from Tedia (USA). Double deionized water was prepared by a Milli-Q water-purification system (Millipore, MA, USA). Dulbecco's Modified Eagle Medium (DMEM), fetal bovine serum (FBS), Phosphate buffer saline (PBS), antibiotics, antimycotics, and trypsin were purchased from Invitrogen (Carlsbad, CA, USA). 2,2-diphenyl-1-(2,4,6-trinitrophenyl) hydrazyl (DPPH) and ascorbic acid were obtained from Sigma Chemicals (St. Louis, MO, USA). 3-(4,5-dimethylthiazol-2yl)-2,5-diphenyltetrazolium Bromide (MTT) was purchased from Invitrogen (Grand Island, NY, USA).

### 2.2. Sample Collection and Authentication

All samples were collected from different growing locations and Hong Kong drug stores. A list of sample collection information is shown in Table 1. Samples from different growing locations (AV5-AV10, AP1-AP2) were authenticated based on herbarium specimen (deposited in the Herbarium of State Key Laboratory of Chinese Medicine and Molecular Pharmacology, Shenzhen, China) and their morphological and microscopic characteristics; while those from Hong Kong drug stores were authenticated based only on their morphological and microscopic characteristics. All samples were identified as the leaves of *Apocynum venetum* L. or *A. pictum* Schrenk.

**Table 1:** A list of *Apocynum venetum* and *Apocynum pictum* sample collected for this study.

Sample Codes	Geographical/Collection Sources	Morphological and Microscopic Characteristics			Species ^b^
		Lateral Vein	Texture	Papillae of Epidermal Cell ^a^	
AV1	Hong Kong Drug stores	distinct	thin, fragile	–	AV
AV2	Hong Kong Drug stores	distinct	thin, fragile	–	AV
AV3	Hong Kong Drug stores	distinct	thin, fragile	–	AV
AV4	Hong Kong Drug stores	distinct	thin, fragile	–	AV
AV5	Tianjin province, China	distinct	thin, fragile	–	AV
AV6	Hebei province, China	distinct	thin, fragile	–	AV
AV7	Shanxi province, China	distinct	thin, fragile	–	AV
AV8	Shanxi province, China	distinct	thin, fragile	–	AV
AV9	Shanxi province, China	distinct	thin, fragile	–	AV
AV10	Shanxi province, China	distinct	thin, fragile	–	AV
AP1	Xinjiang province, China	indistinct	thick, hard	+	AP
AP2	Xinjiang province, China	indistinct	thick, hard	+	AP
AP3	Hong Kong Drug stores	indistinct	thick, hard	+	AP
AP4	Hong Kong Drug stores	indistinct	thick, hard	+	AP
AP5	Hong Kong Drug stores	indistinct	thick, hard	+	AP
AP6	Hong Kong Drug stores	indistinct	thick, hard	+	AP

^a^ +: present, –: absent; ^b^ AV: *Apocynum venetum* L., AP: *Apocynum pictum* Schrenk.

### 2.3. Sample Preparation for Antioxidant Analysis

The extracts prepared for antioxidant analyses were prepared by accurately weighing 10 g fine powder of samples (AV and AP) and mixing with 100 mL distilled water. After shaking in a horizontal shaker at 37 °C, 300 rpm for 2 h, the solution was centrifuged at 3000 rpm for 30 min. The supernatant was collected, and the residue was re-extracted two more times with the same volume of distilled water. Finally, the pooled supernatant was lyophilized by freeze-dryer (LABCONCO FreeZone^®^ 6, Houston, TX, USA). The dried extracts were stored at −20 °C before use.

### 2.4. Fingerprint Analysis by HPLC-DAD-MS

About 0.5 g of accurately weighed sample was sonicated with 20 mL 70% ethanol for 30 min. Then, the mixture was centrifuged at about 3000× *g* for 5 min. Finally, the sample solution was filtered through a 0.45 µm PTFE filter before HPLC-DAD-MS analysis.

Chromatographic analysis was carried out on a Agilent Zorbax column (250 mm × 4.6 mm, 5 μm, Agilent Corp., Wilmington, DE, USA) at 25 °C using an Agilent 1100 liquid chromatography system, equipped with a quaternary solvent deliver system, an auto-sampler and a DAD system. The detection wavelength was set at 360 nm. The gradient elution of the mobile phase consisting of (A) acetonitrile and (B) 0.1% (v/v) formic acid is as follows: 12%–17% of A at 0–10 min; 17% of A isocratic at 10–23 min; from 17% to 20% of A at 23–35 min; from 20% to 30% of A at 35–45 min; from 30% to 70% of A at 45–50 min; 70% of A isocratic at 50–60 min. 15 min re-equilibrium was allowed between injections. The flow rate was 1.0 mL/min and aliquots of 10 μL were injected into the HPLC.

An Agilent MSD Trap VL module mass spectrometer was connected to the Agilent 1100 HPLC instrument via an orthogonal ESI interface. The LC effluent was introduced into the ESI source in a post-column splitting 5:1. Ultra high purity helium (He) was used as the collision gas, while high-purity nitrogen (N_2_) was used as the nebulizing gas. The ionization parameters of negative ion mode were set as follows: ion spray voltage 3.5 kV, nebulizing gas of nitrogen at 30.0 psi; drying gas of 9.0 L/min at 350 °C. Data acquisition was performed in the full scan mode form m/z 100 to 1500 for MS and with an accumulation time of 200 ms and 7 microscans was averaged per recorded scan.

### 2.5. Quantitative Analysis of Hyperoside and Isoquercetrin by HPLC-UV

Quantitative analysis of hyperoside and isoquercetrin in samples were carried out using HPLC-UV method cited in the Hong Kong Chinese Materia Medica Standard [16]. Briefly, 0.2 g sample, accurately weighed, was refluxed with 40 mL 70% methanol for 3 hours. Then, the mixture was centrifuged at about 3000× *g* for 5 min. Afterward, the residue was washed with 8 mL 70% methanol and was centrifuged at about 3000× *g* for 5 min. All extracts was combined and marked to 50 mL volumetric flask. Finally, the sample solution filtered through a 0.45 μm PTFE filter before HPLC-DAD analysis. Chromatographic analysis was carried out on a Agilent Zorbax column (250 mm × 4.6 mm, 5 μm) at 25 °C using an Agilent 1100 liquid chromatography system, equipped with a quaternary solvent deliver system, an auto-sampler and a DAD system. The detection wavelength was 254 nm. The gradient elution of the mobile phase consisting of (A) acetonitrile and (B) 0.05% (v/v) trifluoroacetic acid is as follows: 17%–18% (A) at 0–22 min; from 18% to 35% (A) at 22–30 min; 10 min re-equilibrium was allowed between injections. The flow rate was 1.0 mL/min and aliquots of 10 μL were injected into the HPLC.

### 2.6. Free Radical Scavenging Capacity

The 2,2-diphenyl-1-(2,4,6-trinitrophenyl) hydrazyl (DPPH) assay was used to compare the antioxidant activities of the AV and AP extracts. The two extracts of different concentrations were prepared with ddH_2_O for dilution. Fifty microliters of the aqueous dilutions of the two extracts were mixed with 950 μL DPPH solution (0.024 mg/mL DPPH in absolute methanol) in duplicate. Fifty microliters ddH_2_O was mixed with 950 μL DPPH solution in duplicate as the control. All these mixtures were incubated in the dark for 2 h. For the measurement, absolute methanol was used to set blank at 515 nm. The absorbance values of the control and the aqueous dilutions of AV and AP sample extracts were measured at 515 nm and recorded. The percentage free radical scavenging capacity (SR %) was calculated as (1 − A_Sample_/A_Control_) × 100%. The values of the concentration that scavenged half the quantity of DPPH free radical (SC50) of the two extracts were determined using GraphPad Prism version 5.01 for Windows (GraphPad Software, San Diego, CA, USA, www.graphpad.com).

### 2.7. Cell Culture and Cell Viability Assay

Human embryo liver LO2 cells were cultured in high glucose DMEM medium with 10% FBS (v/v) and 1% Penicillin/Streptomycin (v/v) in a humidified incubator maintained at 37 °C and 5% CO_2_. Cells were subcultured with 0.25% trypsin every three days with a subcultivation ratio of 1:4. The protective effects of the two extracts against H_2_O_2_ induced cell damage were evaluated with MTT cell viability assay. Cells for viability assay were collected by trypsinization. They were seeded in a 96-well plate with 1 × 10^4^ cells in 100 μL complete medium per well and incubated overnight. Then, the culture medium was removed and the seeded cells were treated with or without different concentrations of two extracts dissolved in complete medium and incubated for 2 h in the incubator at 37 °C. After that, the culture medium with AV and AP extracts was changed with complete medium and 300 μM H_2_O_2_ and further incubated for 24 h. After 24 h treatment, the medium with 300 μM H_2_O_2_ was replaced with MTT solution (0.5 mg/mL in serum-free high glucose DMEM medium) and cells were incubated for 3 h. At the end, the MTT solution was removed and 100 μL DMSO was added into each well to dissolve formazan in cells. After complete dissolution, the absorbance was measured at 570 nm, with 655 nm as reference. The percentage cell viability was calculated by: (Absorbance of drug treated group/Absorbance of model group) × 100%.

### 2.8. Statistical Analysis

All biological data are presented as means standard errors of means (S.E.M), and *n* denotes the number of replications for each data point. Statistical analysis was performed by using analysis of variance (ANOVA) to detect significant differences between multiple groups. A value of probability (*p*) < 0.05 was considered to be statistically significant. All statistical analysis tests were performed using GraphPad Prism 5.02 for Windows (GraphPad Software, San Diego, CA, USA). As for chromatographic fingerprint data, all the calculations and chemometrics data treatments were carried out using MATLAB versions 6.5. (Mathworks, Natick, MA, USA).

## 3. Results and Discussion

### 3.1. Chromatographic Fingerprint Studies between AV and AP by HPLC-DAD-MS

**Figure 1:**
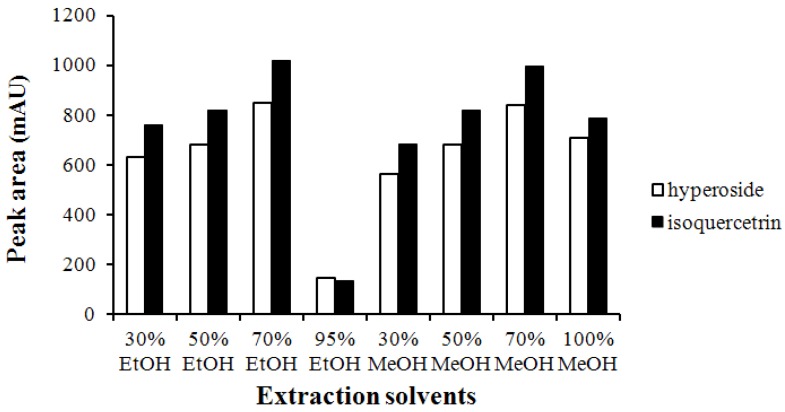
Effect of solvent in extracting hyperoside and isoquercitrin in AV samples.

Prior to the chemical fingerprints comparison between AV and AP, optimization of extraction procedures and chromatographic conditions have to be established. In order to obtain the optimal extraction conditions, the influences of the solvents, extraction methods and durations were investigated while two markers, hyperoside and isoquercetrin [11,17] served as assessment indicators. Different percentage of ethanol (30%–95%) and methanol (30%–100%) were used for the preliminary studies. As a result, shown in Figure 1, 70% ethanol and 70% methanol were found to be the most effective by comparing the peak areas in HPLC-DAD chromatograms. Due to environmental concerns, 70% ethanol was selected as extraction solvent. Sonication and heat-reflux extraction methods were also examined, and both methods brought out similar extraction effects. Because of the easier operation, sonication was selected. Finally, different durations (10–60 min) were carried out and the results suggested that 30 min was optimal. Thus, sonication with 70% ethanol for 30 min was the optimal extracting condition for flavonoids in sample.

In this study, acid was added into the mobile phase to minimize the tailing effect and achieved a better separation between hyperoside and isoquercetrin during the separation process. Formic acid was used in our work because it was applicable in the MS detection with good intensity. In addition, different chromatographic columns including Agilent Zorbax SB C18, ACE C18 and Grace Alltima C18 were tested. Agilent Zorbax was finally selected as it provided a shortened duration in separating the two markers. As for the detection wavelength, 360 nm was utilized as most of the compounds detected in the chromatogram possessed strong UV absorbance.

**Figure 2:**
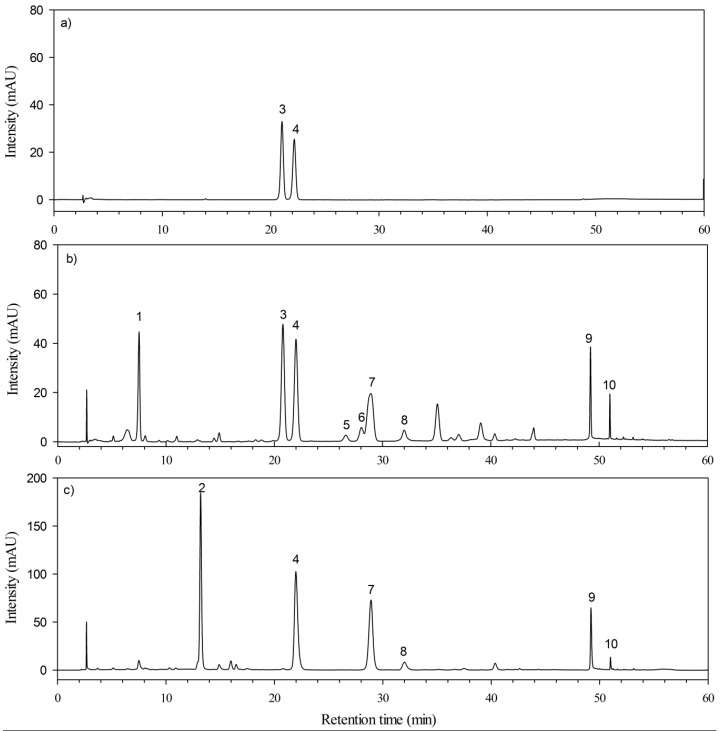
HPLC/UV (366 nm) chromatograms of the studied flavonoids components: (**a**) two flavonoids markers (50 ppm of hyperoside and 60 ppm of isoquercetrin) and (**b**) the extracts of *Apocynum venetum* L.; (**c**) extracts of *Apocynum pictum* : (1) Chlorogenic acid; (2) Rutin; (3) Hyperoside; (4) Isoquercetrin; (5) Acetylated hyperoside; (6) Trifolin; (7) Acetylated isoquercetrin; (8) Astragalin; (9) Quercetin; (10) Kaempferol.

Figure 2 shows typical chromatograms of the standard solution containing the two flavonoids markers (50 ppm of hyperoside and 60 ppm of isoquercetrin) (Figure 2a) and the extracts of AV (Figure 2b) and AP (Figure 2c). The presence of the two flavonoids was confirmed by comparing the retention time and UV spectrum of the corresponding peak with those of the standards. The retention time of hyperoside and isoquercetrin are 20.79 min and 22.00 min, respectively. It was observed that chromatograms of AV and AP were different from each other. Due to the limited chemical information obtained from the UV spectra, LC-MS was adopted to identify the chemical constituents of AV and AP samples. In our work, electrospray ionization (ESI) methods in both negative and positive modes were compared in the detection of flavonoids. Negative mode ESI was found to be more sensitive for flavonoids and further utilized for flavonoids identification. Table 2 summarized the UV and MS data obtained from the chromatograms for AV and AP samples. A total of 10 flavonoids were characterized from the 70% ethanol extracts of the two species, and tentatively identified by comparing their mass spectra with those of reference isolated flavonoids reported in some previous literatures [12,18].

**Table 2:** HPLC-DAD-MS data of major flavonoids identified in the extract of *Apocynum venetum* and *Apocynum pictum*.

Name of Flavonoids	Peak	Retention Time (min)	UV λ_max_	MW	MS Fragmentation Data (ve)
Chlorogenic acid	1	7.50	240, 326	354	352.7, 190.7
Rutin	2	18.94	256, 354	610	608.7, 300.8
Hyperoside	3	20.79	256, 354	464	462.6, 300.8
Isoquercetrin	4	22.00	256, 354	464	462.6. 300.7
Acetylated hyperoside	5	26.59	256, 354	506	504.6, 462.8
Trifolin	6	28.04	264, 346	448	446.7, 283.7
Acetylated isoquercetrin	7	28.92	366, 354	506	504.6, 462.6
Astragalin	8	31.99	266, 346	448	446.6, 283.6
Quercetin	9	49.20	256, 370	302	300.8, 150.7
Kaempferol	10	51.00	266, 366	286	284.8, 150.8

### 3.2. Similarity Index Evaluation and Principal Component Analysis

To examine the chemical composition sameness and difference between AV and AP samples, the entire chromatographic fingerprints were applied and evaluated by similarity index (SI) calculation, recommended by State Food and Drug Administration of China (SFDA) since 2004. Before analysis, it is necessary to correct the retention time shift in all chromatograms obtained because multivariate analysis is highly sensitive to retention time shift among chromatograms. Local least square method [19], which has been successfully applied in chromatographic alignment in quality control of TCM, was utilized in this work.

**Figure 3:**
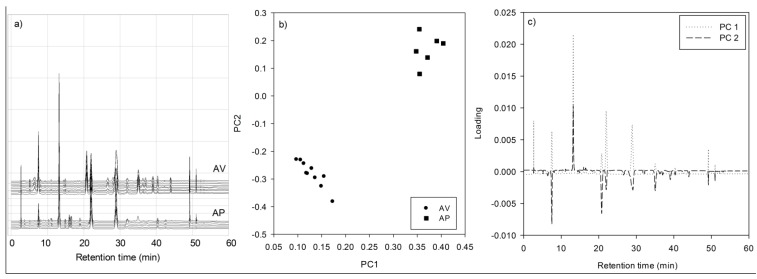
(**a**) HPLC chromatograms of 10 AV samples (upper) and 6 AP samples (lower; (**b**) score plot on the two principal components of HPLC fingerprint data from AV (●) and AP (■) samples; and (**c**) loading plot the PC1 (···) and PC2 (----) for HPLC fingerprint data.

Correlation coefficient is used as a measure for similarity index analysis. The chromatogram of a standardized extract is recommended to serve as the reference profile, but unfortunately it is rarely available. Therefore, the mean or median chromatographic profile of the data set is usually used. The results of correlation coefficient of each chromatogram with the mean HPLC fingerprint of AV and AP are shown in Table 3. As expected, chromatograms of the samples (shown Figure 3a) from the same species showed much better similarity as indicated by the correlation coefficient higher than 0.9. From Table 3, it is obvious that chromatograms of AV are different from AP and can be distinguish from those of the other species. Since AV samples (as well in AP samples) are similar to each other, sample number AV10 and AP2 are selected at random to carry out further antioxidant assay works.

Principal component analysis (PCA), which is chemometrics method commonly used in quality control of TCM, was utilized to investigate the variation within datasets. By looking into the loading and score plots (Figure 3b,c) on the first two principal components, rutin (peak 2) and hyperoside (peak 3) play significant factors in differentiating between AV and AP.

**Table 3:** Results calculated from similarity index method.

Sample Information	SI Value	
	Calculated from AV Mean Chromatograms	Calculated from AP Mean Chromatograms
AV1	0.952	0.347
AV2	0.976	0.379
AV3	0.987	0.314
AV4	0.987	0.347
AV5	0.927	0.394
AV6	0.986	0.350
AV7	0.968	0.348
AV8	0.977	0.355
AV9	0.964	0.316
AV10	0.968	0.312
AP1	0.235	0.954
AP2	0.354	0.997
AP3	0.463	0.961
AP4	0.330	0.993
AP5	0.350	0.994
AP6	0.397	0.988

The contents of hyperoside and isoquercetrin in all AV and AP samples were further carried out according to the assay method of Hong Kong Chinese Materia Medica Standard [16]. Validation of the developed HPLC-DAD method on quantification of hyperoside and isoquercetrin was carried out by linearity, precision, accuracy and stability. All the calibration curves of hyperoside and isoquercetrin exhibited good linearity (*r*2 > 0.999 within test ranges). The limit of detection (LOD) and the limit of quantitation (LOQ) of hyperoside and isoquercetrin were 42.5 ng/mL, 212.5 ng/mL and 39.4 ng/mL, 197.0 ng/mL, respectively. Also, the developed assay method of hyperoside and isoquercetrin had good accuracy with 103.26% ± 1.93% and 101.34% ± 2.43%, respectively, in sample (*n* = 5). Stability was tested with both standard and sample extract stored at room temperature within two days. The repeatability of the extracts was less than 5% and two flavonoids were found to be rather stable. A conclusion could be generalized that this assay showed good reproducibility.

The developed HPLC-UV method was applied to analyze two flavonoids in 10 batches of AV and six batches of AP samples and Table 4 summarized the overall results of the contents of hyperoside and isoquercetrin in all the samples collected. It is obvious that the average content of hyperoside in AV was much larger than that in AP. It is believed that hyperoside could serve as a good chemical marker to differentiate AV and AP samples.

**Table 4:** Content of Hyperoside and Isoquercetrin.

AV Sample	Content of Markers (mg/kg)		AP Sample	Content of Markers (mg/kg)	
	Hyperoside	Isoquercetrin		Hyperoside	Isoquercetrin
AV1	6414	5218	AP1	ND	6289
AV2	6050	5636	AP2	ND	13539
AV3	5542	6015	AP3	ND	17808
AV4	5463	6390	AP4	ND	14987
AV5	5351	5311	AP5	ND	13206
AV6	5894	4406	AP6	ND	14312
AV7	5438	4917			
AV8	5565	4900			
AV9	4232	3483			
AV10	5415	4448			
Mean	5536.4	5072.4	Mean	ND	13356

ND = Not detected.

### 3.3. Comparison between Free Radical Scavenging Capacities

**Figure 4:**
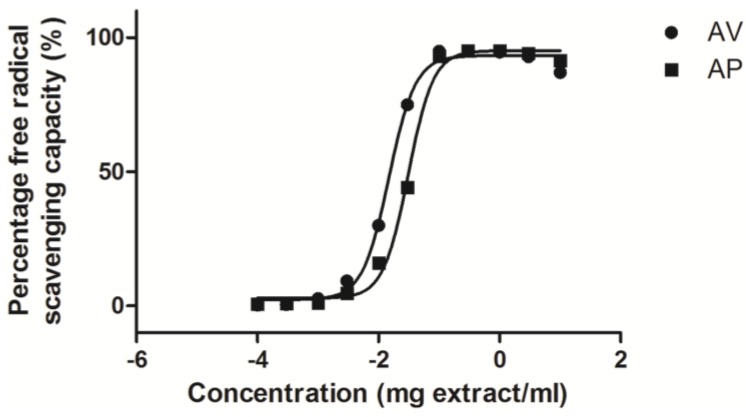
Percentage free radical scavenging capacity (SR %) of AV and AP extracts. Data are expressed as means ± SEM, *n* = 3.

The free radical scavenging capacities of the AV and AP sample extracts were measured with DPPH free radical scavenging assay (Figure 4). The overall free radical scavenging capacities of AV extract was found to be better than that of AP extract (*n* = 3). The SC_50_ values of AV and AP extracts were determined to be 0.01506 ± 0.00085 and 0.03118 ± 0.00084 mg extract/mL, respectively. The SC_50_ value of AV extract was significantly smaller than that of AP extract (*p* < 0.0005), indicating that AV extract provided higher antioxidant activity than AP extract.

Flavonoid compounds have been reported to possess significant antioxidant power [20,21]. In HPLC analysis, the flavonoid profile among these two samples varied tremendously. Additionally, the overall flavonoid content in AV was shown to be higher than that of the AP. AV extract was found to have a significantly higher free radical scavenging activity than AP extract. It may be contributed to by the difference in their overall flavonoid contents.

### 3.4. Protective Effect Against H_2_O_2_-Induced Damage in LO2 Cells

**Figure 5:**
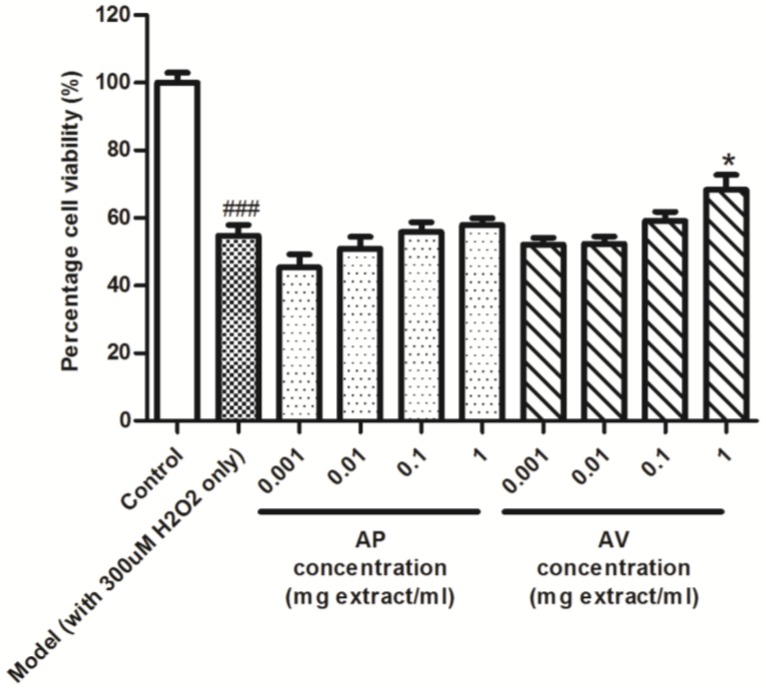
Effect of AV and AP extracts on H_2_O_2_-induced cytotoxicity in LO2 cells. Data are expressed as means ± SEM; *n* = 4. ^###^ *p* < 0.001 represents a significant difference when compared model with the control. * *p* < 0.05 represents a significant difference when compared the AV and AP extracts treated group with model.

It is agreed that the complicated composition of medicinal herbs makes it impossible to estimate the anti-oxidant potency of individual sample with a single method. Additionally, chemical methods may not be able to completely reflect the actual situation in biological condition [22]. Human embryo liver LO2 cells are considered a normal liver cell line. It is commonly used to study the role of oxidative stress in liver [23]. Any protective effects offered by the herbal extracts in LO2 cells could reflect the beneficial efficacy in a more complex and diverse cell populations such as liver. As shown in Figure 5, H_2_O_2_ (300 mM) treatment for 24 h reduced the percentage cell viability of LO2 cells to 54.72% ± 3.24%, and 1 mg extract/mL AV extract (but not AP extract) pre-treatment for 2 h protected LO2 cells against H_2_O_2_-induced damage and increased the percentage cell viability to 68.37% ± 4.41% (*p* < 0.05). Both H_2_O_2_-induced damage in LO2 cells and DPPH free radical scavenging assay concluded that AV possess higher antioxidant activity that AP.

## 4. Conclusions

Our overall results demonstrated that there are significant differences, including chemical constituents and biological activities, between AV and AP. AV is proven to have a better antioxidant activity than AP, both chemically and biologically. In addition, hyperoside could be a suitable chemical marker for discrimination between AV and AP and it is suggested to be used in quality assurance and control of AV.
